# The Impact of Flavonols on Cardiovascular Risk

**DOI:** 10.3390/nu14091973

**Published:** 2022-05-09

**Authors:** Joanna Popiolek-Kalisz, Emilia Fornal

**Affiliations:** 1 Department of Bioanalytics, Medical University of Lublin, 20-090 Lublin, Poland; emiliafornal@umlub.pl; 2 Department of Cardiology, Cardinal Wyszynski Hospital in Lublin, 20-718 Lublin, Poland

**Keywords:** flavonols, antioxidants, cardiovascular prevention, cardioprotection, coronary heart disease

## Abstract

Cardiovascular disease (CVD) is the leading cause of deaths globally. The main target for prevention of cardiovascular (CV) risk are lifestyle changes, including particular dietary recommendations, involving high intake of fruits and vegetables. Flavonols are a subgroup of flavonoids—compounds present in fruits, vegetables, and tea—known for their antioxidative properties. There are many findings about the beneficial impact of flavonols on general CV risk and its factors, but mainly from in vitro and animal model studies. This paper summarizes data from human studies about flavonols’ impact on general CV risk and its factors. A high dietary intake of flavonols could decrease CVD mortality directly or through impact on selected CV factors; however, available data are inconsistent. Nonetheless, specific groups of patients (smoking men, hypertensive and diabetic patients) can potentially benefit from selected dietary modifications or flavonols (quercetin) supplementation. Future investigations about kaempferol and myricetin are needed.

## 1. Introduction

Cardiovascular disease (CVD) is the leading cause of deaths globally [1,2]. Proper cardiovascular (CV) risk assessment is based on specified risk factors. According to the American Heart Association, they include: dyslipidemia, hypertension, cigarette smoking, male sex, elder age, or diabetes [3]. Lifestyle is the most important factor determining health status, and as a result, CVD prevention through lifestyle modification, e.g., dietary approach, is crucial [2]. Recommendations indicate plant-based and Mediterranean diets as the most beneficial diets in CVD prevention [2]. They are based on increased fruit, vegetable, lean protein, and fiber consumption [2]. Vegetables and fruit are the sources of flavonoids, while flavonols are their subgroup distinguished by the chemical structure including a 3-hydroxyflavone backbone. The most important flavonols are: quercetin, kaempferol, myricetin, and isorhamnetin; however, this group also includes morin, galangin, fisetin, kaempferide, azaleatin, natsudaidain, pachypodol, and rhamnazin, which are less prevalent [4]. 

**Figure 1:**
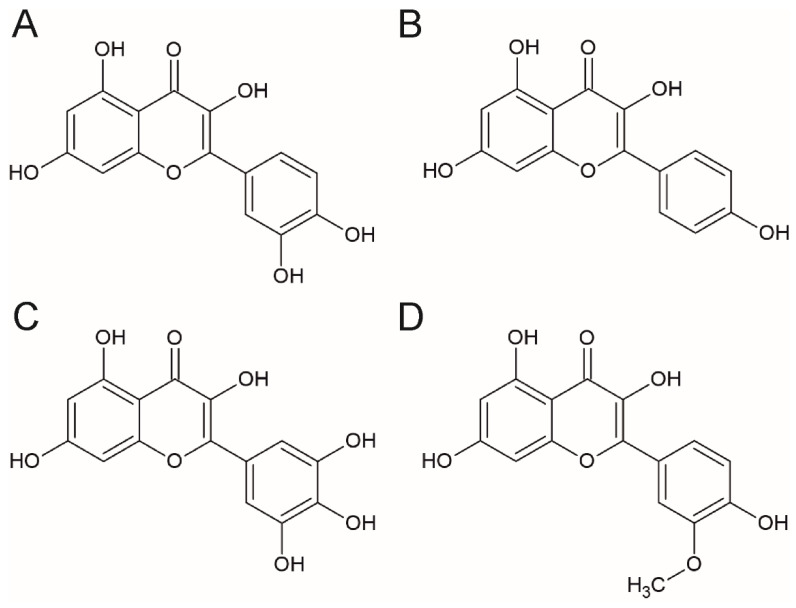
The chemical structure of the selected flavonols (**A**) quercetin, (**B**) kaempferol, (**C**) myricetin, and (**D**) isorhamnetin.

The free forms of flavonols are called aglycones. They share a 3-hydroxyflavone backbone and differ by the presence and position of hydroxyl groups. The number of hydroxyl groups contribute to the potency or bioactivity of these compounds [5]. Quercetin and kaempferol differ by the presence of an additional hydroxyl group at the R1 position in the quercetin molecule. That is why quercetin is less chemically stable and more reactive than kaempferol [5]. Comparing to quercetin, myricetin has an additional hydroxyl group in the R5 position, while isorhamnetin is O-methylated in the R3 position. The structures of the most important flavonols: quercetin, kaempferol, myricetin, and isorhamnetin are presented in Figure 1.

Aglycones are generally unstable and present low solubility due to lipophilicity. These properties lead to glycosylation susceptibility which improves the stability of hydrophobic compounds. There are numerous glycoside forms of flavonols differed by sugar moieties (mainly monosaccharides: glucose, rhamnose, galactose, arabinose, and xylose; and the disaccharide rutinose) and their positions [6,7]. Furthermore, flavonol aglycones and glycosylated forms can be further conjugated, which also impacts their biological and chemical activity as the position of conjugate attachment may block the active hydroxyl sites [8].

The chemical structure of flavonols modifies their activity and also impacts their bioavailability. Aglycons due to their lipophilic properties passively diffuse unmetabolized from the intestinal lumen into the enterocytes [9]. Glycoside forms due to their lipophobic properties must be hydrolyzed into aglycones by the lactase-phlorizin hydrolase enzyme on the intestinal brush border prior to passive absorption [10]. An alternative route involves the sodium-dependent glucose transporter which transports glycosides into the enterocytes where they are hydrolyzed into aglycones by cytosolic β-glucosidase [10]. After, aglycones present in the enterocytes can be either directly absorbed into the blood stream or they can be metabolized before further absorption. The metabolism consists of phase I (oxidation and O-demethylation) and phase II (sulfation, glucuronidation, and methylation) [9]. The metabolized aglycones are then also transported into the blood stream. The absorbed aglycones bound to serum albumin and the metabolites are transported to the liver where they undergo the phase I and II metabolism [11]. Flavonols’ metabolism in body tissues is not well understood.

The major contributors to everyday dietary flavonols intake are: onions, tea, and apples [12]. Other flavonol-rich products are: kale, lettuce, tomatoes, broccoli, grapes, berries, and red wine [4,13]. The most abundant individual flavonol is quercetin, followed by kaempferol, myricetin, and isorhamnetin [13,14,15].

Flavonols are associated with a cardioprotective potential and reduced risk of vascular diseases; however, most of the studies are conducted in vitro or on animal models [4,16,17]. The most investigated link refers to flavonols’ antioxidant and anti-inflammatory activity; however, they can impact the CV system by other numerous pathways, e.g., angiotensin-converting enzyme inhibition [18], antiplatelet aggregation effects [19,20], 3-hydroxy-3-methylglutaryl-coenzyme A reductase degradation, or low density lipoprotein (LDL) receptor expression in the liver [21]. Although these results are promising, data gathered from studies in humans are not abundant [16,17]. Such information, especially from randomized clinical trials, can be helpful and directly lead to more precise dietary or supplementation recommendations in CVD prevention. The aim of this review was to gather available information about flavonols’ impact on recognized CV risk factors in humans.

## 2. Methods

To assess the effects of selected flavonols on cardiovascular risk and its factors, the Medline (http://www.ncbi.nlm.nih.gov/pubmed, accessed on 28 December 2021) was searched using a combination of the following queries in titles and abstracts: (flavonols OR kaempferol OR quercetin OR isorhamnetin OR myricetin) AND (“cardiovascular disease” OR “cardiovascular risk” OR “blood pressure” OR hypertension OR hyperlipidemia OR lipids OR cholesterol OR triglycerides OR “coronary heart disease” OR “myocardial infarction” OR diabetes OR glucose OR smoking). The search was limited to studies performed in humans and included all types of studies (observational, clinical trials, randomized controlled trials, or meta-analyses). There were no time restrictions for the publication time. As a result, 37 papers were retrieved for full-text reading.

## 3. General CVD Incidence and Mortality

### 3.1. Observational Study Data

Dietary flavonols have been widely tested in the context of general CVD mortality; however, the results are inconsistent. The first study that analyzed this issue was the Zutphen Elderly Study from 1993 [22]. Flavonoid intake was significantly inversely associated with coronary heart disease (CHD) mortality [22]. The analyzed flavonols were quercetin, kaempferol, myricetin, but the study was focused on total flavonoid intake without the division into individual compounds [22]. The findings were confirmed after 10 years of follow-up [23]. 

In a mixed Finnish population, an inverse gradient was observed between dietary intake of flavonoids and total mortality, similarly between flavonoids and CHD mortality in patients without CVD at baseline [24]. The relation was significant in women and nonsignificant in men [24]. The study did not investigate the influence of individual flavonoids, but analyzed the participation of different foods. The consumption of apples and onions was inversely associated with total and CHD mortality with a stronger relation in women [24].

In a male population, there was no strong inverse correlation between intake of total flavonoids, quercetin, myricetin, or kaempferol and CHD incidence [25]. However, in the subgroup with pre-diagnosed CHD, a modest but still nonsignificant inverse association between intake of flavonoids and CHD mortality rate was found [25].

Tea as the source of flavonols was examined in the Caerphilly Study. Among Welsh men, flavonol intake, mainly from tea, was not related to CHD incidence, but was weakly related to CHD mortality [26]. The authors suggested that flavonols from tea to which milk is added in this region are probably not absorbed [26].

A 34,492 female population study showed that total flavonoid intake was associated with a decreased risk of CHD deaths in postmenopausal women [27]. Moreover, broccoli intake was also strongly associated with reduced risk of death from CHD [27]. 

In the Rotterdam study, dietary intake of flavonols (quercetin, kaempferol, and myricetin) was significantly inversely associated with fatal myocardial infarction (MI) incidents, but not with all events of MI [28]. Intake also involved drink consumption. The relative risk of any MI incident was lower in patients drinking daily >375 mL of tea compared to non-tea drinkers [28].

Another Finnish study showed that patients with higher quercetin intake had lower mortality from CHD [29]. This relation was not significant for kaempferol and myricetin [29]. It is worth noting that the dietary history did not include consumption of tea, which is also a rich source of flavonols, and therefore it could have possibly interfered with the results. However, the authors stated that tea consumption in Finland is relatively low, and as a result, the expected tea contribution to flavonol dietary intake would be small [29]. The study showed that intake of dietary sources rich in flavonoids such as apples and onions was significantly associated with a decrease in CHD mortality [29].

In the study by Sesso et al., for general incidence of CVD and important vascular events, there was no significant trend observed between flavonoid intake in women [30]. The relation with CVD was not found for any individual flavonol (quercetin, kaempferol, or myricetin) [30]. Among dietary sources, broccoli and apple consumption was associated with reductions in CVD risk, but the relation was not significant [30]. Similarly, daily consumption of ≥4 cups (946 mL) of tea was associated with a reduction in important vascular event risk, but with a nonsignificant trend [30].

A population-based health survey among a mixed elderly population showed that high dietary intake of kaempferol was associated with lower risk of acute MI; however, there was no clear dose–response gradient observed [31]. What is more, there was no significant impact of quercetin or myricetin intake on acute MI incidence observed in this study.

Similar observations were made in Health Nurses’ Study where there was no association between flavonol intake and risk of nonfatal MI or fatal CHD [32]. Higher intakes of individual quercetin, kaempferol, or myricetin were not significantly associated with a lower risk of nonfatal MI. Among them, only higher intake of kaempferol was associated with a lower risk of CHD death, while intake of tea, onions, apples, and raisins or grapes was not significantly associated with the risk of coronary events [32]. Lower risk of coronary events was observed in patients with higher intake of broccoli [32]. It is worth noting that this long-term study consisted of a large (66,360) but only female population. 

### 3.2. Conclusions

Data from observational studies in mixed populations suggest that flavonol dietary intake is inversely associated with CVD death risk; however, it does not confirm such relation with general CVD incidence [22,23,28]. The advantages of this data are large study groups and a long-time observation. On the other hand, these studies were based on questionnaire-collected data and the relation was analyzed mainly for total flavonols without division into single compounds. The intake was also assessed in tertiles, quartiles, or quintiles, not in absolute values, and it may be not accurate enough. Another flaw of analyzed studies is that in some of them, as already mentioned, only general flavonoid intake was analyzed and sometimes, important dietary sources of flavonols were missed [29]. 

Observational studies focused only on groups consisting of one sex did not confirm the trend. In a female population, dietary flavonol intake (analyzed altogether and for individual compounds) was not significantly associated with any CV event (nonfatal or fatal) [24,27,30,32]. In a male population, only patients with previously established CHD presented a modest but still nonsignificant inverse association between dietary flavonol intake and CHD mortality rate [25]. For other combinations of intakes and CV events in a male population, no significant relation was found [24]. 

For individual subgroups, quercetin and kaempferol dietary intakes were associated with lower mortality from CHD [29,32]. What is more, high kaempferol intake was associated with lower risk of acute MI. The relation between other flavonol subgroups and types of events was not confirmed [31]. 

In terms of specific food intake, the relation between tea consumption and CV events was not consistent. In a few studies, high intake of tea was associated with lower relative risk of CV events [28], while this was not found in other studies [26,30]. The differences might result from, e.g., milk addition which interferes with flavonol bioavailability or the exact level of tea consumption [26]. Other foods indicated as possible CVD risk reductors are onions, apples, and broccoli; however, the significance of the results is not consistent among different studies [24,27,30,32].

Presented conclusions from available human studies are consistent with meta-analyses performed in 2003 and 2021 [33,34]. 

The simplified summary of flavonols’ impact on CVD incidence and mortality is presented in Table 1.

## 4. Hypertension

### 4.1. Observational Studies Data

In the course of a large study by Cassidy et al. the aspect on hypertension and flavonoid habitual dietary intake was examined [12]. There was no evidence for the correlation between general flavonoid intake and a reduction in incidents of hypertension. A similar observation was also made for specific flavonols (quercetin, kaempferol, isorhamnetin, and myricetin) [12]. The study was conducted on a large population (156,957 participants); however, it was limited by an observational study design, based on patients’ self-assessment and self-reporting. 

### 4.2. Interventional Study Data

In a double-blind study performed by Conquer et al., 28-day quercetin supplementation did not modify blood pressure (BP) levels [35]. In this study, quercetin plasma concentration was elevated after application of a quercetin-containing supplement; therefore, satisfactory bioavailability was confirmed [35].

Another double-blind placebo-controlled study showed that BP was not altered in prehypertensive patients after quercetin supplementation [36]. However, in patients with first stage of hypertension, it was significantly reduced after quercetin treatment [36]. The small population of this study (41 patients) encourages examination of these findings further on larger group of patients. 

On the other hand, in double-blinded placebo-controlled study by Egert et al., it was reported that daily administration of 150 mg quercetin decreased BP in both the whole study group (metabolic syndrome patients) as well as in the hypertensive subgroup [37]. The bioavailability was confirmed by measurement of plasma quercetin concentration. 

Quercetin supplementation could also be beneficial in healthy male smokers [38]. A double blinded placebo-controlled trial showed that 100 mg quercetin supplementation significantly decreased both systolic and diastolic BP [38]. 

When 1095 mg quercetin was administered to normotensive and the first stage hypertensive male group, the significant reduction of mean BP was observed only in the hypertensive group, even though quercetin plasma concentration in both groups was elevated compared to placebo [39]. The mechanism of this phenomenon is still unknown.

A similar relation was observed in obese-to-overweight patients. Daily administration of onion skin extract powder (equivalent of 162 mg quercetin) decreased 24 h systolic BP in the hypertensive group when compared with placebo [40]. In the total group, including pre-hypertensive patients, quercetin did not significantly affect 24 h BP levels and office BP levels [40].

In a double-blinded randomized clinical trial, daily administration of 500 mg quercetin in type 2 diabetic (T2D) patients decreased systolic BP significantly, but the changes in diastolic BP were not significant [41]. The limitation of this study is that it was conducted only on female participants. 

A double-blind study involving healthy male patients with different apolipoprotein E genotypes showed that daily consumption of 150 mg quercetin led to a decrease in postprandial systolic BP [42].

An interesting approach was presented in a randomized study with 15 healthy volunteers where none of the tested quercetin doses (0, 50 mg, 100 mg, 200 mg, or 400 mg) changed BP values 60 min after administration [43]. 

In male patients with essential hypertension and gout, daily administration of 2000 mg quercetin for 6 months and then 1000 mg quercetin for next six months resulted in an antihypertensive effect (reduction of systolic and diastolic BP by 5.5% and diastolic BP by 3.6%); however, the authors did not present clear statistical analysis to prove significance of the results [44].

In a double-blinded placebo-controlled trial reported by Burak et al., daily administration of 190 mg quercetin with 3.6 g alpha-linolenic acid in healthy volunteers did not change BP values after 8 weeks [45]. 

### 4.3. Conclusions

Dietary flavonol intake was analyzed only in one of the reported studies, where there was no association between hypertension incident reduction and general flavonoid or specific flavonol (quercetin, kaempferol, isorhamnetin, and myricetin) intakes [12].

Available studies on BP reduction were mainly interventional, focusing on quercetin supplementation’s impact on hypertension. They generally showed that quercetin supplementation decreased the BP level in hypertensive patients, while such a relation was not observed in prehypertensive (healthy) patients in most of the studies [36,40,45]. BP reduction was observed in patients with other known CVD risk factors (T2D, metabolic syndrome, smoking, or hyperuricemia) [38,40,41,44]. Only one study confirmed BP reduction in non-hypertensive patients without any other CV risk factors [37]. Other studies have shown BP reduction in healthy male smokers and female T2D patients [38,41]. The advantage of these studies was using a controlled quercetin dose, where the observed relation could be dependent on quercetin dose. However, it is worth noting that bioavailability from artificial sources can differ that from dietary sources. Nevertheless, few studies confirmed their conclusions by testing quercetin levels in plasma [35,37]. Furthermore, the study groups were small, and therefore studies on larger groups should be performed to confirm these observations.

The presented conclusions referring to quercetin’s impact on BP levels are consistent with the results from a meta-analysis from 2016, which showed that a significant reduction in BP level was associated only with quercetin doses ≥500 mg/day [46] and from 2019 when quercetin had an impact only on systolic BP [47].

There are no available interventional studies focused on other flavonols and BP level.

## 5. Dyslipidemia

### 5.1. Interventional Study Data

In the interventional double-blinded study by Conquer et al., quercetin 28-day supplementation (1 g/day) did not modify serum total, LDL cholesterol (LDL-C), or high-density lipoprotein cholesterol (HDL-C) or triglyceride levels in healthy individuals [35]. 

In a double-blind placebo-controlled study, supplementation of flavonol extract (54.1 mg isorhamnetin, 20.2 mg quercetin, and 3.4 mg kaempferol) did not alter TC, HDL-C, LDL-C, or triglycerides in healthy men after 4 weeks [48].

Another double-blinded, placebo-controlled study showed that administration of 150 mg quercetin in metabolic syndrome patients decreased serum HDL concentration, while total cholesterol (TC), triglyceride, and the LDL-C/HDL-C, TC/HDL-C, and triglycerides/HDL-C ratio were unaltered [37]. Nonetheless, quercetin significantly decreased plasma concentrations of oxidized LDL-C, which is known for its atherogenic influence [37].

On the other hand, a double-blinded placebo-controlled study in healthy male smokers showed that 100 mg quercetin supplementation significantly reduced serum concentrations of TC and LDL compared to placebo [38]. Furthermore, significant increases were observed in HDL-C serum concentrations in both groups, however, HDL-C level changes were significantly higher in quercetin supplementation group [38].

Another interesting observation was made among patients with dyslipidemia who received a quercetin supplement. There was observed a reduction of TC, triglyceride and LDL-C values with parallel increase in HDL-C; however, the authors did not reveal the dose of quercetin and did not present any statistical analysis [49].

In diabetic patients 500 mg quercetin daily did not cause significant changes in TC, LDL, triglycerides, and triglycerides/HDL-C and LDL-C/HDL-C ratios [41]. 

In healthy men with different apolipoprotein E genotypes, daily administration of 150 mg quercetin significantly decreased postprandial triacylglycerol concentrations and increased HDL-C level compared to placebo [42].

Moreover, in a study by Burak et al., daily administration of 190 mg quercetin with 3.6 g alpha-linolenic acid for 8 weeks improved lipid profiles in healthy volunteers [45]. Nonetheless, significant decrease in TC and LDL-C was observed in both alpha-linolenic acid + quercetin and alpha-linolenic acid + placebo groups, which suggests a lack of the quercetin’s impact on the lipid profile in this study [45].

### 5.2. Conclusions

The only available quercetin supplementation human studies on cholesterol profile were interventional. There are no studies analyzing kaempferol or myricetin application in terms of dyslipidemia. The mentioned studies used quercetin supplements instead of dietary sources which enabled standardization of the applied quercetin dose.

Most of the studies confirmed that quercetin supplementation could bring some benefits for the lipid profile; however, results describing the exact impact of different lipid fractions are not consistent [37,38,40,41,42,49]. In most of the studies, there was no significant positive influence of quercetin on the lipid profile [35,41]. However, in two studies, in the male population, LDL-C reduction (healthy male smokers) and HDL-C elevation (heathy men with different apolipoprotein E genotypes) were observed [38,42]. It may lead to a conclusion that quercetin supplementation can be beneficial only in selected groups of patients (men). It is a worth noticing that male sex is a CV risk factor.

The meta-analysis results are also inconsistent as the meta-analysis from 2017 did not show any changes in the lipid profile (apart from a significant triglycerides reduction at quercetin doses >50 mg/day) [50] while the meta-analysis from 2020 indicated a significant decrease in TC and LDL-C after quercetin supplementation [51]. 

## 6. Diabetes Mellitus

### 6.1. Observational Study Data

A trend toward a reduction in risk of T2D was associated with higher quercetin and myricetin intakes in the above-mentioned Finnish study by Knekt et al.; however, it was not significant [29]. This relation between myricetin and CHD mortality was not maintained [29]. 

In a large observational study on a group of 38,018 women free of CVD and diabetes, dietary intake of quercetin, kaempferol, and myricetin was not significantly associated with T2D risk [52]. Moreover, in 344 nondiabetic women from this study, total intake of flavonols was not significantly related to plasma concentrations of fasting insulin or glycated hemoglobin [52]. However, women consuming ≥1 apple/day showed a significantly reduced T2D risk compared with those who did not consume apples [52]. An analogical trend was observed for tea, as tea consumption was also inversely associated with diabetes risk but with a borderline significant trend ≥4 cups/day vs. none [52]. 

In a Chinese population, a study analyzing quercetin dietary intake revealed that quercetin intake was inversely related to the prevalence of T2D [53].

### 6.2. Interventional Study Data

In healthy male smokers, daily supplementation of 100 mg quercetin significantly decreased glucose concentrations after 10 weeks [38]. Meanwhile, a 4-week double-blind placebo-controlled study showed that consumption of flavonol extract (54.1 mg isorhamnetin, 20.2 mg quercetin, and 3.4 mg kaempferol) did not change glucose levels in healthy male nonsmokers [48].

In another 4-week placebo-controlled clinical trial, daily administration of a supplement containing 50 mg myricetin three times per day in T2D female patients significantly reduced fasting plasma glucose [54]. 

Moreover, regular consumption of 250 mg of patented blend of chlorogenic acid, myricetin, and quercetin lowered the acute glycemic impact of foods, but also chronically decreased blood glucose levels in a T2D mixed population [55]. 

A meta-analysis of nine randomized controlled trials analyzed the effect of quercetin supplementation on glycemic control among the patients with metabolic syndrome and related disorders [56]. It showed that in a subgroup with a duration of ≥8 weeks and with daily dose of quercetin ≥500 mg, fasting plasma glucose was significantly reduced [56]. Moreover, in a subgroup of patients aged <45 years with the same dose, a significant reduction in insulin concentrations was observed [56]. 

### 6.3. Conclusions

Observational data regarding flavonol intake and T2D risk are not consistent. Generally, there is no significant relation between quercetin, kaempferol, or myricetin dietary intake and T2D incidence [29,52]. The positive observation was made only for quercetin intake in a Chinese population [53]. However, tea and apple consumption, which are good sources of quercetin, was associated with lower diabetes risk in women [52]. This relation might be the result of other compounds present in these products, e.g., flavanols in tea [57].

Interventional studies showed that supplementation of quercetin and myricetin could decrease glucose levels [38,54,55]. These findings are consistent with the meta-analysis which revealed in 2019 that supplementation ≥500 mg/day of quercetin reduced plasma glucose levels, although in the above-mentioned studies, the applied dose of quercetin was lower than 500 mg [56]. 

As presented above, there is inconsistency between data from observational and interventional studies. The reason might be that observational studies observe dietary intake which is lower than supplementation doses. Moreover, the analyzed end-points of the observation differed between types of the studies (diabetes incidence vs. glucose level). Another reason for this disjunction might originate from the study groups which are smaller in interventional studies compared to observational studies. To clarify these differences, more studies are needed.

## 7. Cigarette Smoking

### 7.1. Observational Study Data

It was observed that in Finnish male smokers, intake of flavonols and flavones was inversely associated with nonfatal MI [58]. Moreover, there was also an association with CHD death among these patients, although attenuated [58]. 

### 7.2. Interventional Study Data

Smokers can also benefit from other quercetin supplementation-related results such as cholesterol profile improvement, BP reduction, and glucose level decrease, which was discussed in previous paragraphs [38].

### 7.3. Conclusions

The data from observational and interventional studies are consistent and show that smokers are the group of patients that can particularly benefit from high dietary intake of quercetin or its supplementation [38,58]. It is interesting that quercetin’s positive impact on CV risk factors is present in this group in contrast to other groups (e.g., healthy volunteers). It might be the result of antioxidative properties of flavonols, which can be greatly present in smokers. However, as presented above, the data about quercetin’s influence on CV risk in smokers are limited, and therefore more studies in this area are crucial. Moreover, there are no reports regarding kaempferol or myricetin’s role in smokers in terms of CV risk.

The simplified summary of flavonols’ impact on selected CV risk factors is presented in Table 2.

## 8. Limitations

The main advantages of this study are also its limitations. This paper summarized data from different types of studies, and therefore it cannot be treated as a meta-analysis. Observational studies referred to everyday dietary intake, which can be easily applied in terms of future nutritional recommendations. Observational studies are generally performed on large groups with possibility of long-term follow-up. However, dietary intake of selected products or compounds in this type of studies is usually based on questionnaires, and any deficiency of them (e.g., omitting some products) or mistakes while filling in by patients can interfere with the results regarding investigated intake. Moreover, self-reported questionnaires can analyze mainly clear end-points (e.g., mortality), and they are not useful to observe modifications of subtle risk factor backgrounds. On the other hand, interventional studies are performed with standardized doses, but the influence of non-dietary source intake on bioavailability cannot be excluded. Moreover, the used doses are difficult to maintain in an everyday diet, so the results from this type of studies can lead to supplementation recommendation instead of diet modification.

## 9. Summary and Conclusions

Data from studies regarding flavonols’ impact on CV risk factors in humans are inconsistent. One of the reasons is different approach combining advantages and disadvantages of each type of study that was described in detail in the previous paragraph. The compromise between this data could be made by conduction of an interventional study regarding dietary modifications; however, these kinds of studies are difficult due to standardization and compliance issues. 

Nonetheless, data from available studies suggest a positive impact of flavonol intake on CV risk factors, especially in smokers, hypertensive and T2D patients, or CVD deaths. Regarding other CV risk factors, the correlation was not found. Moreover, most of the studies referred to general flavonol intake or only to quercetin. The data about other flavonols in humans are limited. This area of interest seems promising, although it needs careful and standardized examination in the future. 

## Figures and Tables

**Table 1:** The simplified summary of flavonols’ impact on CVD incidence and mortality.

Study Group	Compound or Group	Outcome	Significant Association
men aged 65–84 years [22,23];	flavonoids	CHD mortality	yes
postmenopausal women aged 55–69 years [27];			
healthy women aged 30–69 years [24]			
men aged 40–75 years with CHD [25];	flavonoids	CHD mortality	no
healthy men aged 30–69 years [24]			
healthy men aged 45–59 years [26]	flavonols	CHD mortality	no
healthy women aged 30–55 years [32]	kaempferol	CHD mortality	yes
healthy mixed population [29]	kaempferol and myricetin	CHD mortality	no
healthy mixed population [29]	quercetin	CHD mortality	yes
healthy women aged 30–55 years [32]	flavonols	nonfatal MI, CHD mortality	no
healthy subjects aged ≥ 55 years [28]	flavonols	fatal MI	yes
healthy subjects aged ≥ 55 years [28]	flavonols	all events of MI	no
healthy women aged 30–55 years [32]	quercetin, kaempferol, myricetin	nonfatal MI	no
healthy men aged 45–59 years [26]	flavonols	CHD incidence	no
men aged 40–75 years [25];	flavonoids, quercetin, kaempferol, myricetin	CHD incidence	no
healthy women [30]			
healthy mixed population aged 65–99 years [31]	kaempferol	acute MI	yes
healthy mixed population aged 65–99 years [31]	quercetin, myricetin	acute MI	no

CVD—cardiovascular disease; CHD—coronary heart disease, and MI—myocardial infarction.

**Table 2:** The simplified summary of flavonols’ impact on selected CV risk factors.

Study Group	Compound or Group	Outcome	Significant Association
Hypertension			
healthy mixed population [12]	flavonoids, quercetin, kaempferol, isorhamnetin, and myricetin	hypertension incidence	no
healthy mixed population [35];	quercetin	BP level	no
prehypertensive patients [36];			
healthy volunteers [45];			
normotensive male patients [39]			
pre-hypertensive obese-to-overweight patients [40]			
first stage of hypertension [36];	quercetin	BP level	yes
metabolic syndrome patients (with and without hypertension) [37];			
hypertensive obese-to-overweight patients [40];			
first stage hypertensive male patients [39];			
healthy male smokers [38]			
female T2D patients [41]	quercetin	systolic BP level	yes
female T2D patients [41]	quercetin	diastolic BP level	no
healthy male patients with different apolipoprotein E genotypes [42]	quercetin	postprandial systolic BP level	yes
male patients with essential hypertension and gout [44]	quercetin	systolic and diastolic BP level	unknown
healthy volunteers [43]	quercetin	BP level 60 min after administration	no
Dyslipidemia			
healthy mixed population [35]	quercetin	LDL-C, HDL-C, TC, and triglycerides	no
metabolic syndrome patients [37]	quercetin	HDL-C, oxidized LDL	yes
metabolic syndrome patients [37]	quercetin	TC, triglyceride, and LDL-C/HDL-C, TC/HDL-C, triglycerides/HDL-C ratio	no
diabetic patients [41]	quercetin	TC, LDL-C, triglycerides, triglycerides/HDL-C, and LDL-C/HDL-C ratio	no
healthy male smokers [38]	quercetin	TC, LDL-C, HDL-C	yes
healthy men with different apolipoprotein E genotypes [42]	quercetin	postprandial triglycerides and HDL-C	yes
patients with dyslipidemia [49]	quercetin	LDL-C, HDL-C, TC, and triglycerides	unknown
healthy volunteers [45]	quercetin	TC and LDL-C	no
healthy male nonsmokers [48]	quercetin, kaempferol, isorhamnetin	TC, LDL-C, and HDL-C	no
Diabetes			
healthy mixed population [29]	quercetin, myricetin	T2D risk	no
women free of CVD and diabetes [52]	flavonols, quercetin, kaempferol and myricetin	T2D risk, fasting insulin, glycated hemoglobin	no
Chinese population [53]	quercetin	T2D prevalence	yes
healthy male smokers [38]	quercetin	glucose concentration	yes
patients with metabolic syndrome and related disorders [56]	quercetin	fasting plasma glucose	yes
T2D female patients [54]	myricetin	fasting plasma glucose	yes
T2D mixed population [55]	myricetin, quercetin, chlorogenic acid	acute glycemic impact of foods, chronic blood glucose	yes
patients with metabolic syndrome aged <45 years [56]	quercetin	insulin concentration	yes
healthy male nonsmokers [48]	quercetin, kaempferol, isorhamnetin	glucose concentration	no
Smoking			
healthy male smokers aged 50–69 years [58]	flavonols	nonfatal MI	yes
healthy male smokers [38]	quercetin	glucose concentration	yes
healthy male smokers [38]	quercetin	BP level	yes
healthy male smokers [38]	quercetin	TC, LDL-C, HDL-C	yes

BP—blood pressure; CV—cardiovascular; HDL-C—high-density lipoprotein cholesterol; LDL-C—low-density lipoprotein cholesterol; MI—myocardial infarction; T2D—type 2 diabetes; and TC—total cholesterol.

## Data Availability

Not applicable.

## Abbreviations

BP	Blood pressure
CV	Cardiovascular
CVD	Cardiovascular disease
CHD	Coronary heart disease
HDL-C	High-density lipoprotein cholesterol
LDL-C	Low-density lipoprotein cholesterol
MI	Myocardial infarction
T2D	Type 2 diabetes
TC	Total cholesterol
